# Adaptive Physiological and Morphological Adjustments Mediated by Intestinal Stem Cells in Response to Food Availability in Mice

**DOI:** 10.3389/fphys.2018.01821

**Published:** 2019-01-08

**Authors:** Isaac Peña-Villalobos, Ignacio Casanova-Maldonado, Pablo Lois, Pablo Sabat, Verónica Palma

**Affiliations:** ^1^Departamento de Ciencias Ecológicas, Facultad de Ciencias, Universidad de Chile, Santiago, Chile; ^2^Laboratorio de Células troncales y Biología del Desarrollo, Departamento de Biología, Facultad de Ciencias, Universidad de Chile, Santiago, Chile; ^3^Center of Applied Ecology and Sustainability (CAPES), Pontificia Universidad Católica de Chile, Santiago, Chile

**Keywords:** caloric restriction, intestinal crypts, mTORC1, jejunum, environmental stochasticity, starvation

## Abstract

Several studies have evaluated plastic changes in the morphology of the digestive tract in rodents subjected to caloric restriction or restricted availability. Nevertheless, studies that link these morphological responses to physiological consequences are scarce. In order to investigate short-term plastic responses in the intestine, we acclimated adult *Mus musculus* (BALB/c) males for 20 days to four distinctive treatments: two caloric regimens (*ad libitum* and 60% of calorie ingestion) and two levels of periodicity of the regimens (continuous and stochastic treatment). At the end of the treatment we analyzed the cell proliferation and cell death dynamics of small intestinal crypts in these animals. In addition, we measured organ masses and lengths, hydrolytic digestive enzyme activities, and energy output from feces. Finally, in order to explore the metabolic changes generated by these dietary conditions we assessed the catabolic activity (i.e., enzymes) of the liver. Our results show that individuals acclimated to a continuous and 60% regimen presented longer intestines in comparison to the other treatments. Indeed, their intestines grew with a rate of 0.22 cm/day, generating a significant caloric reduction in the content of their feces. Besides, both mass and intestinal lengths were predicted strongly by the stabilization coefficient of BrdU+ proliferating cells per crypt, the latter correlating positively with the activity of n-aminopeptidases. Interestingly, by using pharmacological inhibition of the kinase mammalian target of rapamycin complex 1 (mTORC1) by Rapamycin, we were able to recapitulate similar changes in the proliferation dynamics of intestinal stem cells. Based on our results, we propose that the impact of caloric restriction on macroscopic variation in morphology and functional changes in digestive n-aminopeptidases occurs through synchronization in the proliferation rate of stem and/or progenitor cells located in the small intestinal crypts and requires mTORC1 as a key mediator. Hence, we suggest that an excessive stem and progenitor activity could result in increased crypts branching and might therefore underlie the reported intestinal tissue expansion in response to short-term caloric restriction. Summarizing, we demonstrate for the first time that short-term caloric restriction induces changes in the level of cell proliferation dynamics explaining in part digestive tract plasticity in adaptive performance.

## Introduction

What an animal eats defines its biological success (Bozinovic and Martínez del Río, 1996). Diet selection is associated with phenotypic traits ranging from cellular to ecological and evolutionary ones (Karasov and del Rio, 2007; Withers et al., 2016). In this vein, animals must cope with complex and changing food environments in time and space. Consequently, adaptation to varying nutritional environments depends on the temporal pattern of environmental changes—animals should make an association between a food type and their chemical properties—and their behavioral and physiological phenotypic traits.

The digestive tract represents the functional link between foraging and diet selection (energy/nutrients intake) and energy management and allocation. Furthermore, the digestive tract comprises one of the more expensive tissues of the body, in terms of energy and proteins biosynthesis. Thus, it is expected that the animal's “ability” to regulate the amount of digestive tissue changes due to external and internal conditions, which should have deep implications on their performance (Naya et al., 2008). Consequently, animals adjust their digestive morphological and physiological features to cope with changes in food availability/quality during fasting, reproduction, at low ambient temperatures, or to cope with set of combined energy demands (Sabat et al., 1995; Naya et al., 2008; Zhang et al., 2016; Bełzecki et al., 2018).

Flexibility in rodent digestive size occurs in both laboratory and wild species; both mice and rats are representative models of the digestive flexibility capacity of wild species (Naya et al., 2007). To date, several studies have explored intestinal effects of environmental food variability (e.g., caloric restriction, dietary stochasticity, alternate-day fasting, starvation, among others). Nevertheless, their results provided somewhat conflicting evidence for the existence of morphological changes in the organ, probably determined by the intensity or duration of experimental treatments. For instance, some studies indicated no change or reduction in the mass of intestine in mice and rats under caloric restriction (CR), or effects in morphological traits in villous and crypt under CR (Albanes et al., 1990; Yilmaz et al., 2012; Igarashi and Guarente, 2016; Higashizono et al., 2018). However, opposite effects were reported in many rodent species, both under laboratory and natural conditions (laboratory rats; *M. musculus* [C57BL/6J, HsdCpb, BALB/c]; wild rodent species [*Cricetulus barabensis, Akodon azarae, Eothenomys miletus*]). There, a CR, stochastic regimen, starvation or low quality diet resulted in the growth of the intestine mass or increased area or length of microvilli in small intestine. Besides, high-fat diets showed statistically significant decreases in the measures of proliferation in the colonic mucosal (Waheed and Gupta, 1997; Dunel-Erb et al., 2001; Basson et al., 2004; del Valle et al., 2006; Cao et al., 2009; Zhao and Cao, 2009; Zhu et al., 2014). Thus, paradoxically, these rodents possibly assign energy and biomolecules to the increase of an organ responsible for the processing and absorption of food, despite the reduction in the quality or quantity of food.

Along these lines, Yilmaz et al. (2012) reported the effects of CR at the cellular level of the small intestine crypts. These authors proposed a mechanism in which changes in the organismal nutritional state of rodents are sensed by a kinase, called mammalian target of rapamycin complex 1 (mTORC1). A reduced kinase signaling in turn affects the secretory Paneth cell behavior, leading, through their secretion of a paracrine factor (Cyclic ADP-ribose), to an increment in the number of proliferating stem cells (SC) located nearby in the intestinal crypts. More recently, Igarashi and Guarente (2016), found evidence that even though mTORC1 is downregulated in Paneth cells, mTORC1 activity is increased in the SC pool and cooperates with the NAD dependent protein deacetylase SIRT1 to foster the expansion of gut adult SC under CR.

Regarding intestinal growth, a recent study by Langlands et al. (2016) proposed that crypt fission (the division of a single crypt into two daughter crypts) is at the core of normal growth and maintenance of the intestinal tissue. They demonstrated that development of the intestine would occur by either bifurcation or trifurcations in the crypts. In this process, growth, mediated by an increment in cell number (increase in both Paneth cells and SC between fission events), would precede crypt fission. Thus, cellular proliferation could provide a framework to understand not only homeostatic responses, but also explain intestinal tumorigenesis or the organ recovery from injury (Langlands et al., 2016). Consequently, as counteracting processes, crypt fission and fusion could regulate crypt numbers during the lifetime of a mouse (Bruens et al., 2017). Hence, we propose that environmental energy constraint (i.e., environmental food availability) is a factor that determines the proliferation of SC's (and consequently their differentiation) in the intestine, inducing its plastic growth.

Several studies have attempted to understand the impact of CR on organism fitness. Indeed, it has been described that under CR, mitochondria from hepatocytes showed less oxygen consumption (Lambert and Merry, 2004; Lopez-Lluch et al., 2006) eventually negatively affecting the function of mitochondrial electron transport chain complexes such as the cytochrome c oxidase (COX) or complex IV. These metabolic adjustments may be critical for increased health (Jové et al., 2014). Besides, there is early and robust evidence that a CR condition increases the lifespan in virtually all organisms, from yeast to mammals (Masoro et al., 1991; Boily et al., 2008). CR promotes longevity hypothetically by preserving SC and progenitor cell function (McCay et al., 1935; Nakada et al., 2011). However, the literature is missing an integrative analysis of the intestinal responses to environmental food availability that could indicate an adaptive response for the abovementioned paradoxical adjustment.

From our point of view, it is important to understand, from the bottom up, the process that allows the development of these plastic changes in the intestine. Undoubtedly, this understanding would allow us to have an integrative perspective of phenotypic plasticity, useful for both macroscopic and cellular research. Thus, the aim of the present study was to pursue an integrative analysis (from cellular to biochemical and morphological levels) to explain changes in small intestines of mice, acclimated to contrasting dietary conditions. The main questions in this study are: (i) What kind of dietary conditions induce short-term paradoxical intestinal plasticity?; (ii) By which way are SC proliferation processes associated with macroscopic variations in morphology, and what is the temporality of these processes?; (iii) What are the consequences of changes in cellular proliferation dynamics in terms of the digestive capacities?; (iv) Which metabolic changes are generated in the liver by restricted dietary conditions, taking into account that the liver performs several functions in the time of subsequent absorption (e.g., postprandial thermogenesis, glucose storage, lipid metabolism), and finally, (v) Does mTORC1 signaling serves as a downstream mediator of CR? To address these questions, we analyzed the cellular response of the intestinal epithelium (balance between cell death and cell proliferation) in mice, subjected to four treatments in which we varied both the periodicity of feeding and the dietary caloric content across 20 days. In addition, we studied the changes in intestinal mass and function, through the analysis of digestive enzymes (maltase, sucrase, n-aminopeptidase) and the energy content of feces. We also evaluated differences in the metabolic capacities of the liver by assaying the enzymatic activity of cytochrome c oxidase (COX), in order to explore the metabolic changes generated by dietary conditions. In addition, we aimed to determine the rate of size change in the intestine exposed to a reduced dietary availability, by analyzing the intestine growth at three and nine days of dietary acclimation. Finally, in an attempt to understand if intestinal changes in proliferation dynamics were dependent on mTORC1 function, we developed a treatment with Rapamycin (mTORC1 inhibitor).

## Materials and Methods

### Animal Acclimation

Forty-four adult males (3 months old) of the BALB/c strain of *Mus musculus* were obtained from the central animal housing facilities at the Faculty of Sciences. All animal procedures were in accordance with the Chilean legislation and were approved by Institutional Animal Care and Use Committees at the Universidad de Chile and CONICYT.

All our experiments were performed in two rounds, in consecutive months, i.e., at the first opportunity with 24 individuals, and repeated with 20 animals. All data were pooled. Mice were separated into four groups of either six (1st round) or five (2nd round) animals, considering no siblings in the same group. They were housed individually in polypropylene mesh-floor cages without wood flakes, and kept in a temperature-controlled room, maintained at 25° ± 1°C in a LD = 12:12 cycle, with water *ad libitum*. Mice had no access to their feces nor to the small pieces of food (< 5 mm^3^).

Before the experimental treatments, we measured the maximum food intake under the described experimental conditions (i.e., special cages without wood flakes, with wastage of food, and individual housing). From those analyses, we established that food consumption reaches a maximal value of ~70 g per week, despite the general literature that suggests lower values but determined under different conditions (see Table 1). Next, four experimental groups were acclimated during 20 days under the following conditions: (1) continual *ad libitum* food (C-100 treatment), with 10 g of dry food per day; these individuals could eat up to 70 g per week. (2) Continual feeding with restriction at 60%, with 6 g of dry food per day (C-60 treatment); these individuals could eat up to 42 g per week; (3) stochastic alimentation at 100% (S-100 treatment), with three random days of alimentation with 23 g; these individuals could eat near to 70 g per week. Finally, (4) stochastic alimentation with restriction at 60% (S-60 treatment) with 3 days of alimentation with 14 g; these individuals could eat near to 42 g per week. All groups were fed with dried pellets of a commercial food (Prolab RMH 3000, Labdiet, USA). The measured and estimated (in terms of energetic metabolism) ingestion of food by rodents is presented in Table 1.

**Table 1:** Summary of food provision and food intake values.

**Treatment**	**Food provision (g/day)**	**Food intake range (g)**	**Theoretical requirements range (g)**
C-60	6	5.40–4.02	4.96–4.10
S-60	14^*^	10.5–5.28	5.08–3.83
C-100	10	7.37–3.69	5.00–3.86
S-100	23^*^	10.74–4.60	4.88–3.83

Theoretical requirements were determined from metabolic estimates by mean of the body mass (as indicated in Nagy et al., 1999) and assuming a metabolizable energy of commercial pellet of 3.16 kcal/g (Prolab RMH 3000). C-100, continuous alimentation ad libitum; C-60, continuous alimentation with 60% of the ad libitum offer; S-100, stochastic alimentation ad libitum; and S-60, stochastic alimentation with 60% of the ad libitum offer. Asterisk (

* *) indicate groups that fed 3 days by week, randomly assigned*.

After completing the experiments involving 20 days of acclimation, we decided to repeat the CR experiment exclusively with the C-60 group, in order to examine the temporality of changes. In particular, we focused on the proliferation dynamics of the individuals at day 3 and 9 of acclimation.

### Intestine Processing and Analysis

**Figure 1:**
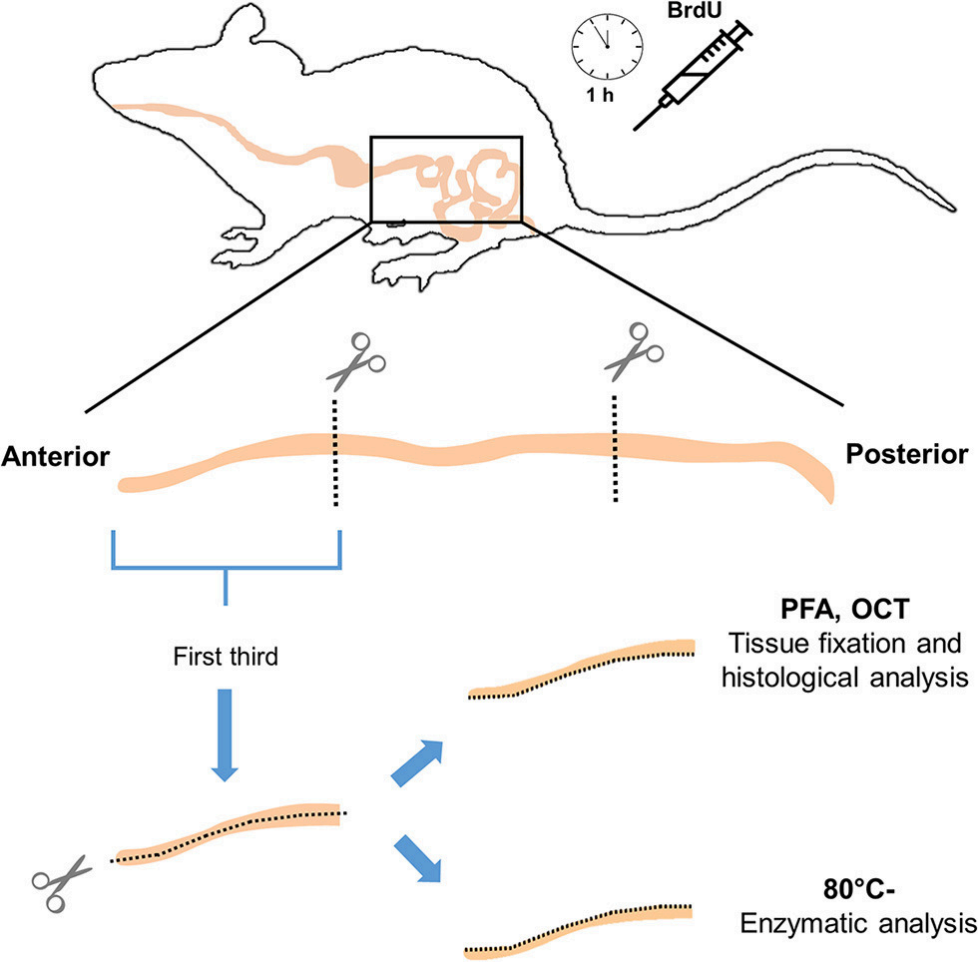
Schematic cartoon illustrating small intestine processing and functional assays of the study (enzymatic activities and proliferation analysis).

After 20 days of acclimation, all individuals were injected intraperitoneally with 800 μL of BrdU 20 mg/mL, (Sigma-Aldrich; reconstituted in NaOH 0.007 N and 0.9% PBS). After 1 h, animals were sacrificed by cervical dislocation and all the digestive tract was removed onto a cooled surface immediately, and organs were weighed (± 0.001 g; Analytical Balance, AUX Series, Shimadzu Scientific Instruments). The intestine was extracted, and then the content was gently removed mechanically, followed by the recording of mass and length (± 0.001 g and 0.1 cm, respectively). Next, the intestine was cut longitudinally, saving one half of the first third for later enzyme assays at −80°C (Thermo Scientific Forma 7000 Series), and the other half for a histological analysis (Figure 1). The segment chosen for histology was fixed in PFA (Sigma-Aldrich) at 4%, during 2 h at 4°C, and then dehydrated overnight in sucrose (Merck) solution at 30%. The tissues were then included in OCT (Tissue-Tek, USA) into disposable vinyl specimen molds (Tissue-Tek Cryomold). The pieces of OCT were cut into 14 μm thick slices by using a cryostat. Additionally, other organs (i.e., stomach, liver, heart, spleen, and gonads) and fat deposits (i.e., brown adipose tissue or BAT, epididymal, and inguinal fat pads) were weighed (± 0.001 g.). The livers were stored at −80°C for further enzyme assays.

### Histological Analysis

Slides were washed for 5 min in PBT (PBS + 0.1% Triton, Calbiochem). The endogenous peroxidase activity was inhibited under darkness for 5 min using a solution of methanol and 0.3% H_2_O_2_. Samples were then washed with PBT and an epitope-unmasking protocol was performed by immersing the samples in a 10 mM citrate/1 mM EDTA buffer solution (pH 6) for 1 h at 80°C and then for 2 min in a solution of 0.1% NaBH_4_. Next, in order to denature the DNA, the slides were immersed in 2 N HCl at 37°C for 30 min and neutralized with 0.1 M Na_2_B_4_O_7_ (pH 8.5) for 5 min. Samples were then blocked for 1 h using serum from an ABC kit (R.T.U Vectastain) and incubated in either monoclonal mouse Anti-Bromodeoxyuridine Clone Bu20a (1: 100; Dako) primary antibody, Anti-Cleaved Caspase-3 (1: 100; Cell Signaling) or Anti-phospho-Histone H3 (1: 100; Merck Millipore) antibody overnight at 4°C. Subsequently, the excess primary antibody was washed and the secondary antibody kit ABC (R.T.U Vectastain) was used according to the manufacturer's instructions. Finally, after washing with PBT, the presence of the secondary antibody was revealed by 1:10 DAB (diaminobenzidine) for 6 min. After this procedure, the nuclei were stained with hematoxylin (Merck Millipore) for 30 s and the sections were dehydrated using a battery of alcohols and mounted with Entellan medium (Merck Millipore).

### Analysis of Histology Samples

Cells were quantified by counting the number of marker-positive cells per crypt, analyzing fields at 100X magnification (Olympus microscope B51), on 36 random crypts per individual, from at least three independent samples from only three individuals of each treatment, randomly selected. Two independent subjects performed the counts.

### Enzyme Assays

#### N-aminopeptidase

Tissues were thawed and homogenized for 30 s in an Ultra Turrax T25 homogenizer at 20,000 rpm, in 20 volumes of a 0.9% NaCl solution. The enzymatic activity in tissue homogenate was measured in order to avoid underestimation of activity. The N-aminopeptidase assay was performed using L-alanine-p-nitroaniline as the substrate. Briefly, 7 μL of homogenate, diluted with a 0.9% NaCl solution, was mixed with 67 mL test mixture (2.04 mM L-alanine-p-nitroanilide in 0.2 M NaH_2_PO_4_/Na_2_HPO_4_, pH 7). The reaction was incubated at 37°C and stopped after 10 min using 126 μL of 2 M cold acetic acid, and the absorbance was measured at 384 nm. The protein content of the intestinal homogenate was determined using the Bradford method. Then, the enzymatic activity was standardized per gram of gut tissue per gram of protein (Sabat et al., 1998), and the activities were presented as standardized hydrolytic activity UI g^−1^ wet tissue and UI mg^−1^ protein (where UI = Hydrolyzed mole min-1).

#### Disaccharidases Maltase and Sucrase

The activity of disaccharidases maltase and sucrase were determined according to the method of Dahlqvist (1964), modified by Martínez del Rio (1990). Briefly, 7 μL of homogenized tissue was incubated at 37°C with 7 μL of disaccharide solutions (maltose or sucrose) 56 mM in 0.1 M Maleate/NaOH buffer, pH 6.5. After 10 min, the reaction was stopped by adding 186 μL of a stop and developing solution (one bottle of GODPAD (Valtek) in 250 mL 0.1 M TRIS/HCl, pH 7). The absorbance was measured at 505 nm with a Thermo Scientific Multiskan GO spectrophotometer after 18 min at 20°C. Standardized enzymatic activities were calculated based on absorbance. The protein content of the intestinal homogenate was determined using the Bradford method. Enzymatic activity was standardized per gram of gut tissue per gram of protein (Sabat et al., 1998), and the activities were presented as previously described.

### Cytochrome C Oxidase

Liver tissues were thawed, weighed, and homogenized in 10 volumes of phosphate buffer 0.1 M with EDTA 0.002 M (pH 7.3) with an Ultra Turrax homogenizer (20,000 rpm) on ice to avoid enzymatic reactions. Samples were then sonicated at 130 watts for 20 s at 10 s intervals, 14 times each, using an Ultrasonic Processor VCX 130, while maintained on ice. Cellular debris was removed by centrifugation for 15 min at 12,000 g and 4°C. The supernatant was carefully transferred into a new tube, avoiding co-transference of the upper lipid layer present in the liver preparations. Protein concentration of the samples was determined by the method described by Bradford (1976), using bovine serum albumin as standard. Activities of cytochrome c oxidase (COX; E.C. 1.9.3.1) were determined spectrophotometrically according to Moyes et al. (1997), with slight modifications. Enzyme activity was determined in 10 mM Tris/HCl pH 7 containing 120 mM KCl, 250 mM sucrose, and cytochrome c reduced with dithiothreitol to a final volume of 0.2 ml. The decrease in D.O. at 550 nm was monitored in a Thermo Scientific Multiskan GO spectrophotometer at 25°C. Enzyme activity in units per gram of wet tissue was calculated using an extinction coefficient of 21.84 mM^−1^cm^−1^ at 550 nm for COX. CS activities were measured according to Sidell et al. (1987) with small modifications.

### Feces Analysis

In order to use the energy values of feces as a proxy of digestive and absorptive capacities (Boily et al., 2008; Yen et al., 2009), their caloric content was determined by combusting dry samples at the end of the acclimation in a Parr 1261 bomb calorimeter (Parr Instruments, Moline, IL). The results are expressed as cal/g in a dry-mass basis.

### Intraperitoneal Injection of Rapamycin

Rapamycin (Calbiochem) was dissolved in dimethyl sulfoxide (DMSO; 5 mg/125 μL) and then diluted in PBS (1: 100 v/v). The total volume was aliquoted in 1 mL and frozen at −20°C until use. Three *ad libitum* fed BALB/c male mice received every 48 h for 20 days an intraperitoneal injection of 100 μL (2 mg/ kg) of the drug. Other three individuals were used as sham group (DMSO vehicle injection).

### Statistics Analysis

Morphological and biochemical data for the different groups were compared using an ANOVA and ANCOVA (with body mass as the covariate) tests. In cases when morphological and physiological variables were correlated with body mass (hereafter referred to as bm), we also used the residuals of those variables against bm to perform Pearson correlations.

The distribution of marker positive cells (i.e., BrdU, Cleaved Caspase 3, or P-Histone 3), was analyzed by mean comparison of their distributions, employing the Kolmogorov-Smirnov comparison of two data sets, with a Bonferroni correction (Peña-Villalobos et al., 2018).

In order to assess the variability in the number of positive labels between crypts in statistical analyses, we calculated the stabilization coefficient (SCo) for each individual, which is the reciprocal of the coefficient of variation (i.e., mean divided by standard deviation; Liu and Zheng, 1989). Thus, a higher value of SCo, implies a tissue with crypts that respond more homogeneously (e.g., there is low variability in the number of marked cells per crypt). All statistical analyses were performed using the STATISTICA statistical package for Windows, and “R” version 3.1.2. for Windows.

## Results

### Macroscopic Morphology and Organ Masses

Given the importance of understanding the impact of the different diet regimens to intestinal physiology and overall on the individual, we initiated our analysis by measuring animals bm. No significant differences among treatments were found in bm at the beginning of the acclimation [*F*_(3,44)_ = 0.194, *p* = 0.90]. However, at the end of the experiment, the S-60 group reduced its bm, respect to the S-100 group [*F*_(3,44)_ = 4.367, *p* = 0.009], indicating the effect of the CR in a stochastically condition. Interestingly, we found that individuals with greater bm experienced after the treatments a more pronounced mass reduction, both in the S-60 and S-100 groups (regression between initial bm and change of mass: *r* = −0.831; *p* = 0.0029; *r* = −0.611; *p* = 0.035, respectively), indicating the use of reserves mainly in stochastics treatments.

Regarding the food intake per treatment, we show that individuals under stochastic regimens eat up to double the food consumed by continuous treatments and their theoretical requirements (see Table 1). Besides, we show great variability in food consumption within all treatments. For instance, within the same group we can find differences of up to six grams of food intake per day per individual (e.g., S-100).

**Figure 2:**
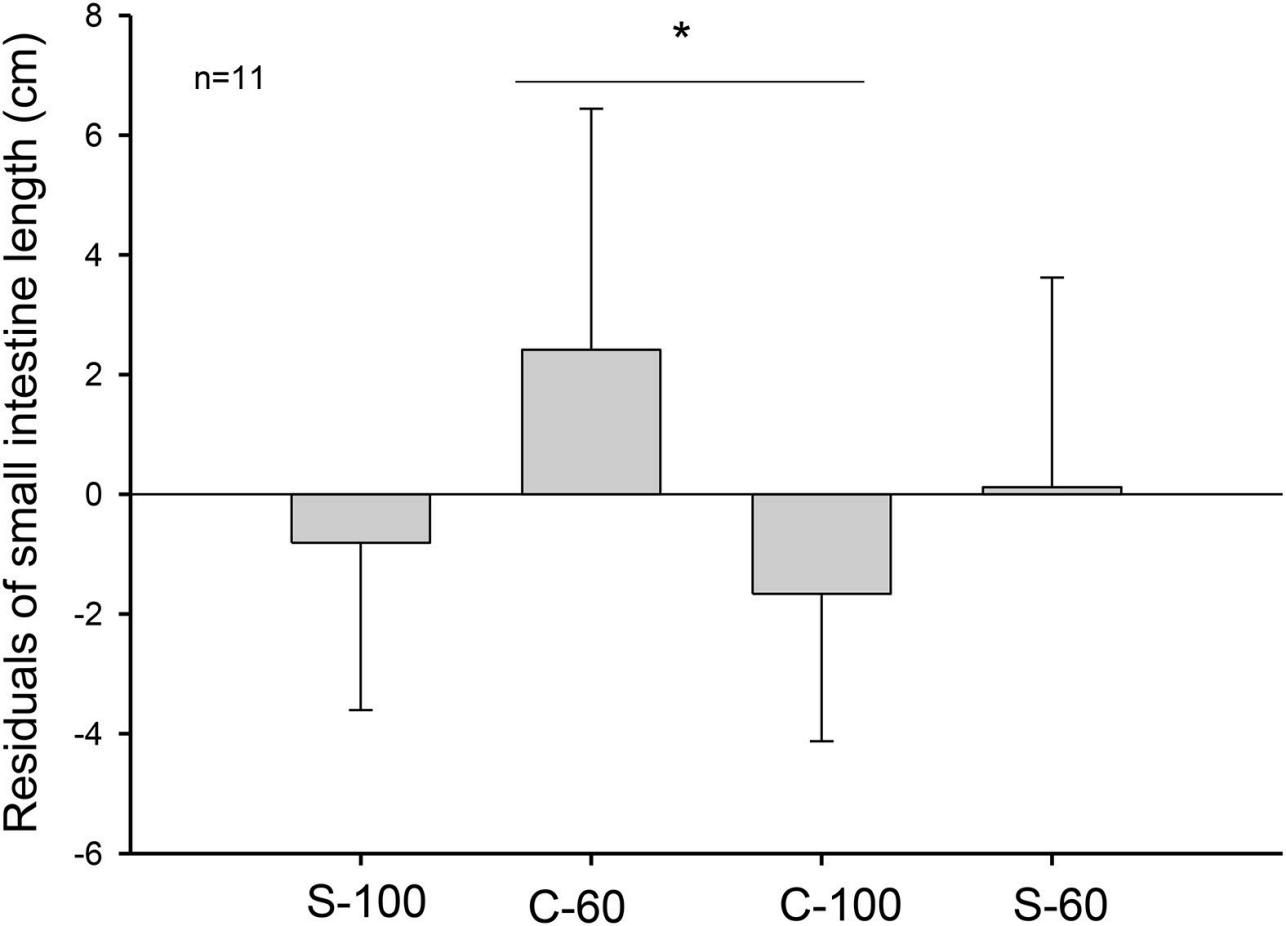
Length of intestines in adult males of *M. musculus* (BALB/c) after 20 days of acclimation to four different treatments. The graph shows 11 individuals per group. C-100, continuous *ad libitum*; C-60, continuous at 60%; S-100, stochastic *ad libitum*; S-60, stochastic at 60%. Asterisk (^*^) denotes differences in Tukey test.

**Figure 3:**
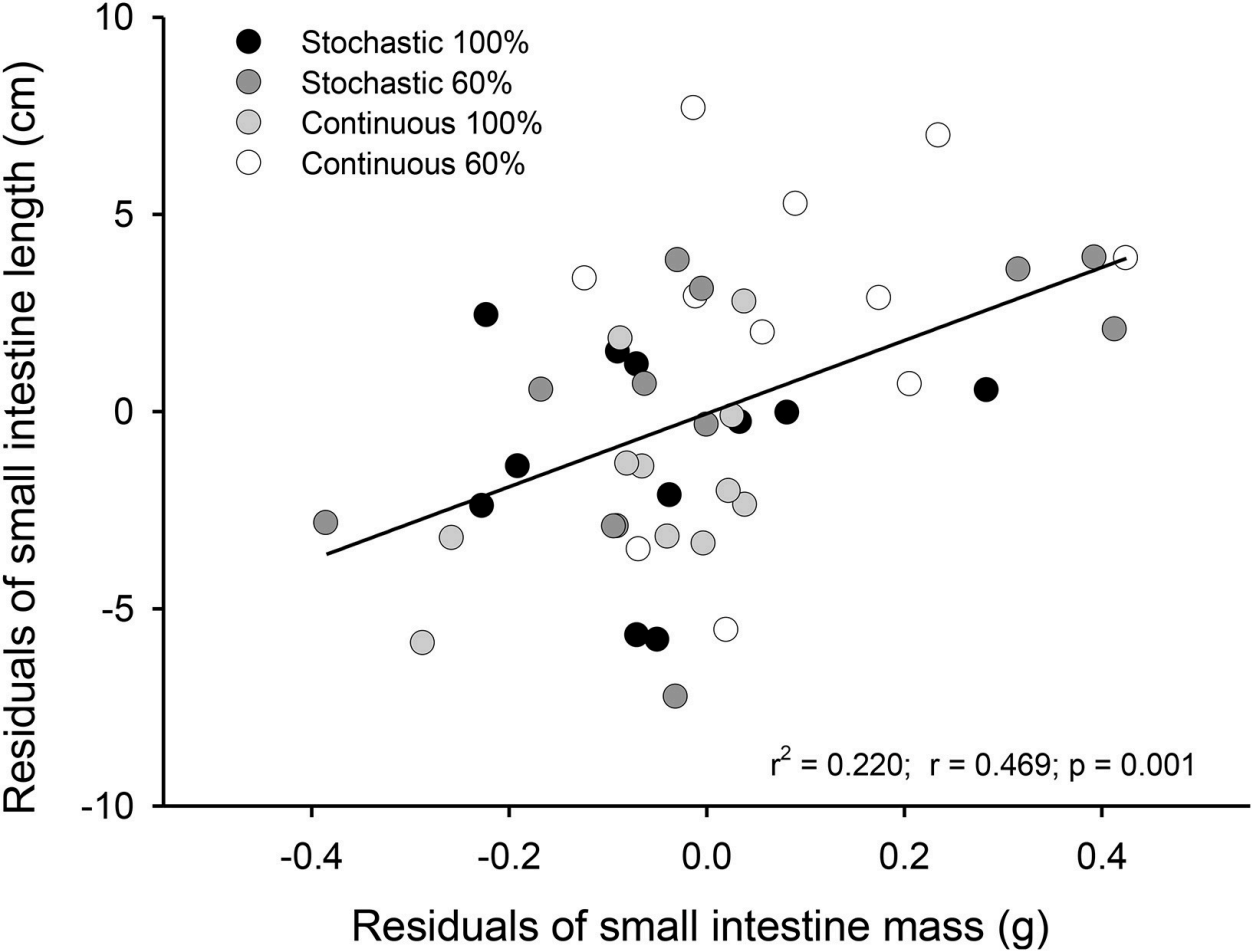
Representation of the relationship between the residuals of mass and residuals of lengths from small intestines of adult *Mus musculus* acclimated 20 days to four treatments; two types of caloric regimens; and two levels of periodicity of the regimens. Specifically, C-100, continuous *ad libitum*; C-60, continuous at 60%; S-100, stochastic *ad libitum*; S-60, stochastic at 60%.

Experimental treatments lead to profound morphological changes in the intestinal epithelium in the C-60 group. When analyzing intestinal size after 20 days of acclimation, we found that C-60 treated intestines were ~4 cm longer (6 to 10%) than intestines exposed to the C-100 condition [ANCOVA *F*_(3, 41)_ = 3.250, *p* = 0.031, Figure 2]. The factorial analysis of residuals of intestinal mass indicates that individuals with 60% of the maximum intake exhibited larger intestines [ANOVA *F*_(1, 41)_ = 4.807, *p* = 0.034]. The residuals of mass and length of intestines correlated positively, with mass accounting for 20% of the length variability (*r*^2^ = 0.220; *p* = 0.001, Figure 3).

Interestingly, we also found a significant effect of the CR treatments on the mass of other organs (Table 2). Mice fed *ad libitum* and continuously (C-100), exhibited the lowest accumulation of inguinal adipose tissue and less accumulation of epididymal adipose tissue when compared to the C-60 and S-100 groups. Besides, the C-100 group had larger reproductive organs than the S-60 group, and the C-100 presented a reduction in the stomach mass in comparison to C-60. Finally, the C-100 displayed a greater spleen mass in comparison to animals acclimated to S-60.

**Table 2:** Organ masses and ANCOVA analysis of *Mus musculus* males, acclimated to four different dietary regimens.

**Variables (g)**	**C-100**	**C-60**	**S-100**	**S-60**	***F*_**(3, 39)**_**	***p***
Body mass *F*_(3, 42)_	28.166 ± 2.618	29.289 ± 4.182	28.114 ± 1.978	26.558 ± 2.573	1.703	0.181
Heart	0.181 ± 0.019	0.207 ± 0.061	0.193 ± 0.022	0.12 ± 0.045	0.870	0.464
Liver	1.512 ± 0.209	1.573 ± 0.261	1.408 ± 0.189	1.409 ± 0.293	0.891	0.454
Epididymal adipose tissue	0.316 ± 0.117*a*	0.488 ± 0.306*b, c*	0.531 ± 0.158*b*	0.366 ± 0.193*a, c*	5.407	0.003
Inguinal adipose tissue	0.205 ± 0.085*a*	0.444 ± 0.294*b*	0.354 ± 0.128*b*	0.367 ± 0.205*b*	6.640	0.001
Reproductive system	0.754 ± 0.108*a*	0.690 ± 0.077*a, b*	0.701 ± 0.068*a, b*	0.648 ± 0.087*b*	2.981	0.042
Stomach	0.194 ± 0.027*a*	0.255 ± 0.060*b*	0.214 ± 0.031*a, b*	0.226 ± 0.057*a, b*	4.301	0.010
Intestine	1.072 ± 0.122	1.239 ± 0.167	1.087 ± 0.156	1.158 ± 0.238	1.980	0.132
Colon	0.363 ± 0.050	0.364 ± 0.068	0.346 ± 0.052	0.371 ± 0.068	0.326	0.806
Caecum	0.209 ± 0.036	0.204 ± 0.027	0.207 ± 0.050	0.205 ± 0.064	0.102	0.958
Kidneys	0.505 ± 0.058	0.517 ± 0.070	0.52 ± 0.043	0.499 ± 0.047	0.271	0.846
Spleen	0.134 ± 0.036*a*	0.117 ± 0.027*a, b*	0.109 ± 0.022*a, b*	0.092 ± 0.013*b*	4.451	0.009

*C-100, continuous alimentation ad libitum; C-60, continuous alimentation with 60% of the ad libitum offer; S-100, stochastic alimentation ad libitum; and S-60, stochastic alimentation with 60% of the ad libitum offer. Letters denote differences in a posteriori test (p < 0.05)*.

### Cellular Proliferation and Turnover

**Figure 4:**
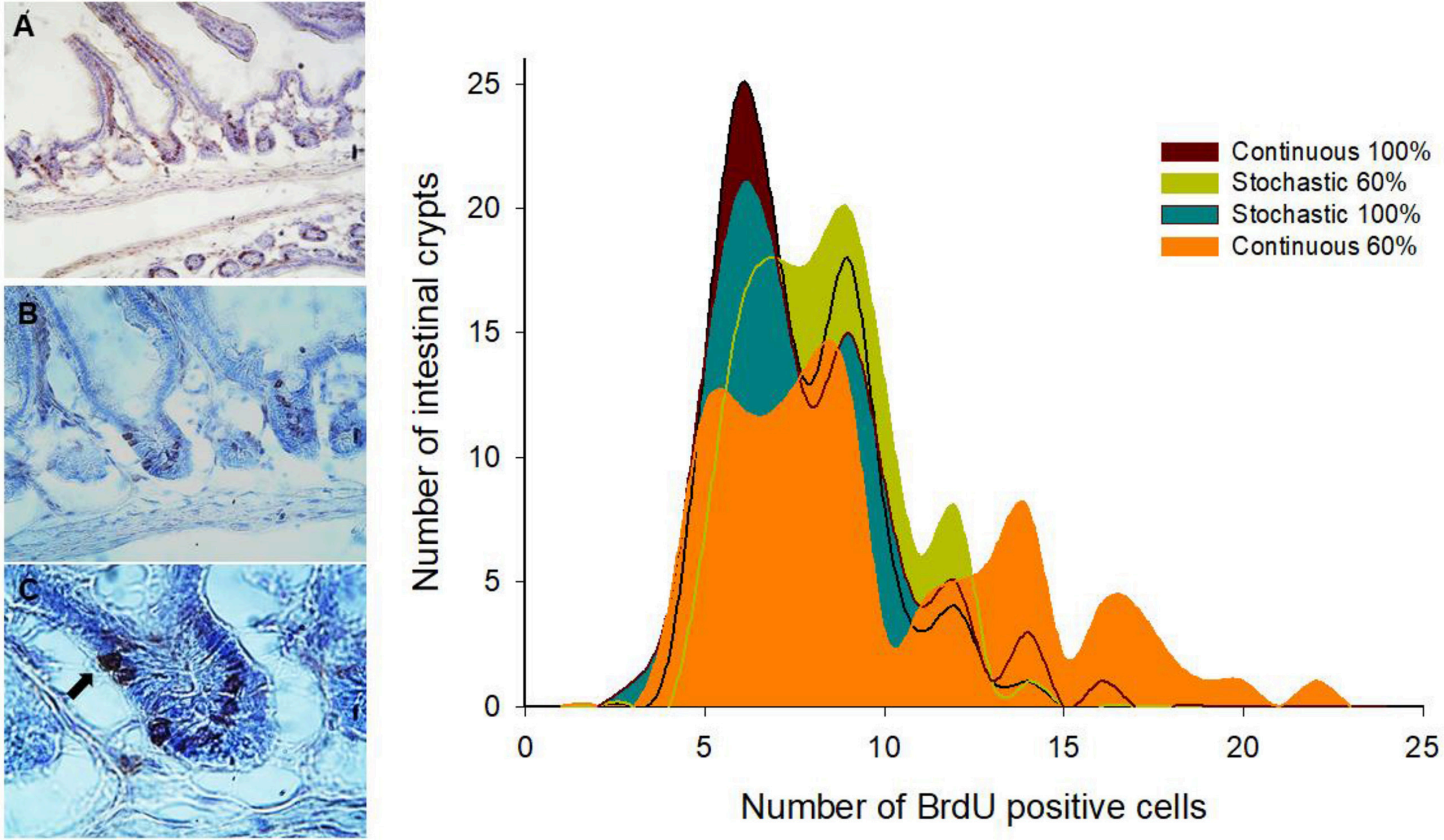
Left side: Representative images of immunohistochemical staining performed on longitudinal sections of the small intestine. BrdU + cells are observed at three optical magnifications: **(A)** 200X, **(B)** 400X, and **(C)** 1000X. The arrow indicates a BrdU + cell individualized in a crypt. Right side: Histogram of absolute frequencies of intestinal crypts indicating numbers of BrdU + cells from adult males acclimated to four different treatments. See text for details.

SC in the crypt of the mammalian intestine are constantly producing new cells, which differentiate into a number of different cell types throughout adulthood. The average number of BrdU+ cells per crypt was not affected by CR treatment [ANOVA *F*_(3, 8)_ = 0.570, *p* = 0.650]; that is, all groups presented a similar average of proliferating cells per crypt. However, an analysis of the distribution of BrdU+ cells per crypt revealed that the C-60 group exhibited a distinctive distribution in comparison to the other groups (Kolmogorov-Smirnov α < 0.008; Table 3). Overall, the histogram depicts five peaks or clusters of proliferating cells, which were conserved in all groups. However, a significant reduction in the first peak (grouping 5-7 BrdU+ cells/crypt) in the C-60 set, with respect to all others, is evident. Moreover, a greater frequency change, shifting in the distribution of the proliferating cell population to higher values corresponding to 7–10, 12–14, and 15 to 20 of BrdU+ cells/crypt, could be also observed in this group (Figure 4).

**Table 3:** Comparison of distributions of cells BrdU+ per crypts, as displayed under four different dietary regimens, by means of a Kolmogorov-Smirnov comparison of two data sets, considering a Bonferroni correction over the *p*-values (differences in α < 0.0083).

**Treatments**	**Continuum 60%**	**Stochastic 60%**	**Continuum 100%**	**Stochastic 100%**
Continuous 60%		0.002	0.001	0.008
Stochastic 60%	0.002		0.062	0.125
Continuous 100%	0.001	0.062		0.996
Stochastic 100%	0.008	0.125	0.996

**Figure 5:**
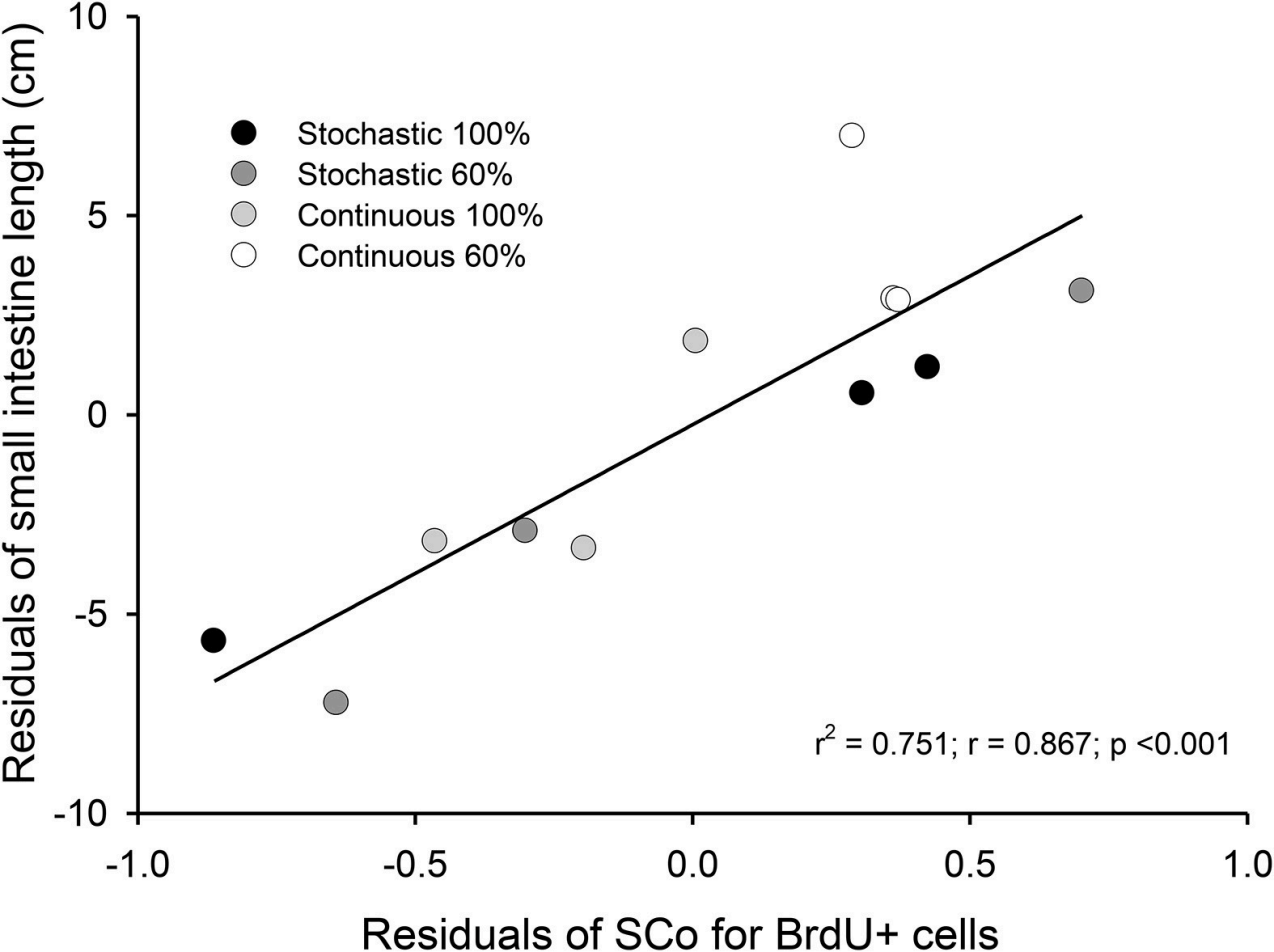
Correlation between residuals of intestine length and the coefficient of stabilization of the number of BrdU + cells per intestinal crypt. We analyzed 36 crypts from three individuals, randomly selected from each group. The samples come from intestines of adult *Mus musculus* acclimated 20 days to four treatments; two types of caloric regimens (*ad libitum* and 60%) and two levels of periodicity of the regimens (continuous and stochastic treatment). Specifically, C-100, continuous *ad libitum*; C-60, continuous at 60%; S-100, stochastic *ad libitum*; S-60, stochastic at 60%.

Regarding the value of SCo, we did not find differences between treatments [ANOVA *F*_(3, 8)_ = 1.095, *p* = 0.406]. However, we found a positive and significant association between the bm and SCo for BrdU+ cells (*r*^2^ = 0.436; *r* = −0.66; *p* = 0.019). In addition, we found a positive relation between the residuals of intestine length and the residuals of SCo for the BrdU+ cells (*r*^2^ = 0.751; *r* = 0.867; *p* < 0.001, Figure 5).

Because changes in BrdU incorporation do not necessarily reflect a change in the proportion of proliferating cells and instead could reveal alterations in the relative lengths of the different phases of the cell cycle, we also evaluated the mitotic marker P-Histone 3. No significant change in the average number of P-Histone 3+ cells per crypt could be observed [*F*_(3, 8)_ = 0.707, *p* = 0.574]. Interestingly, the SCo of P-Histone 3 + cells showed a negative relationship with the spleen and the reproductive system mass (*r*^2^ = 0.556; *p* = 0.009 and *r*^2^ = 0.401; *p* = 0.027, respectively). Moreover, residuals of SCo were positively associated with the residual of BAT and presented a trend with residual mass of the total adipose tissue (*r*^2^ = 0.345; *p* = 0.044 and *r*^2^ = 0.271; *p* = 0.083, respectively). Finally, when evaluating cell death we found that SCo of the Cleaved Caspase 3 + cells also presented a positive correlation with the residual of intestine mass (*r*^2^ = 0.415; *p* = 0.024), suggesting a coordinated increment of proliferation and cellular death in longer intestines.

### Digestive Capacities

**Figure 6:**
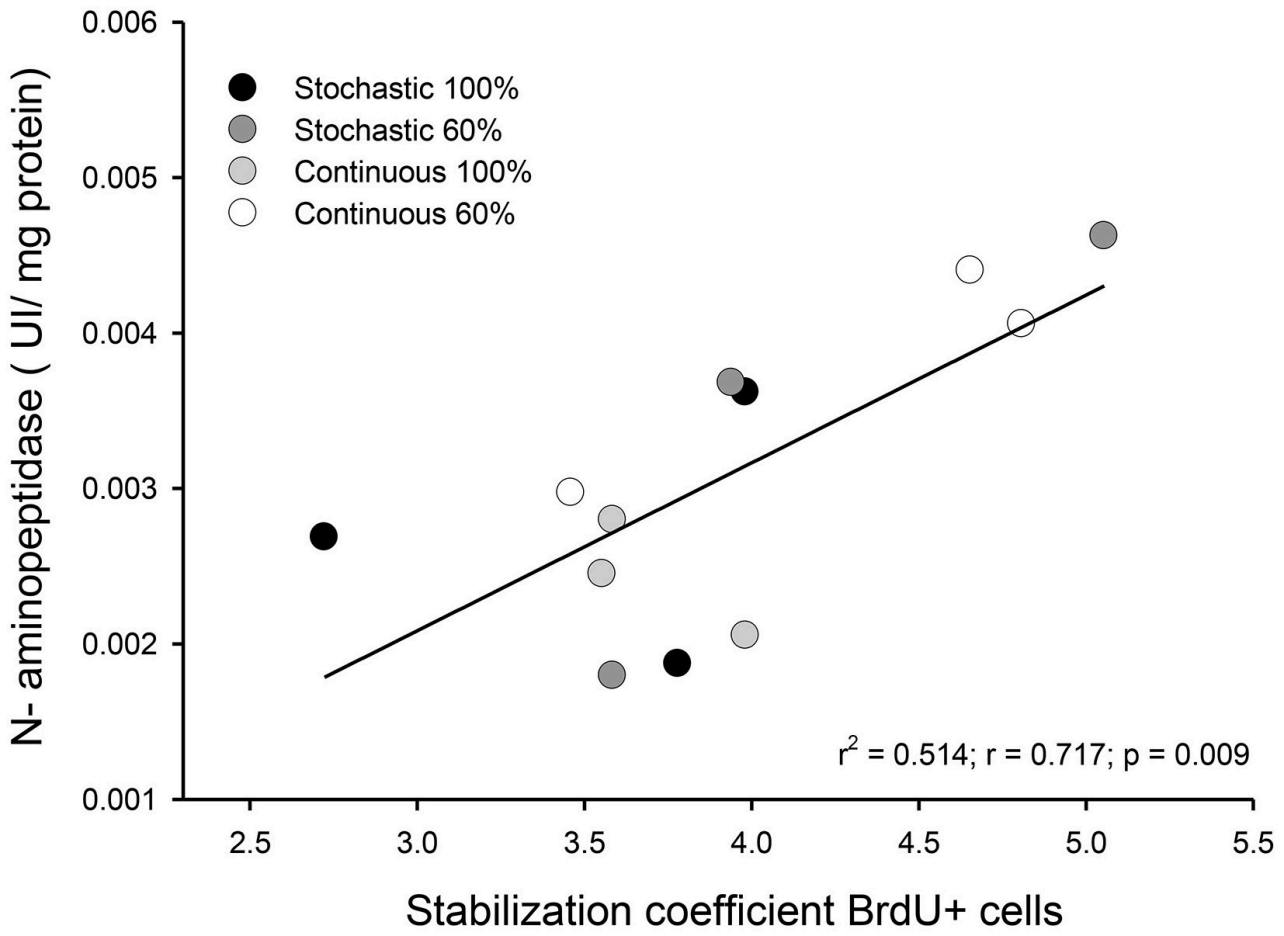
Graph indicating the observed relationship between intestinal n-aminopeptidase activity and the coefficient of stabilization of the number of BrdU + cells by intestinal crypt after being under indicated treatments for 20 days. We analyzed three individuals, randomly selected from each group.

**Figure 7:**
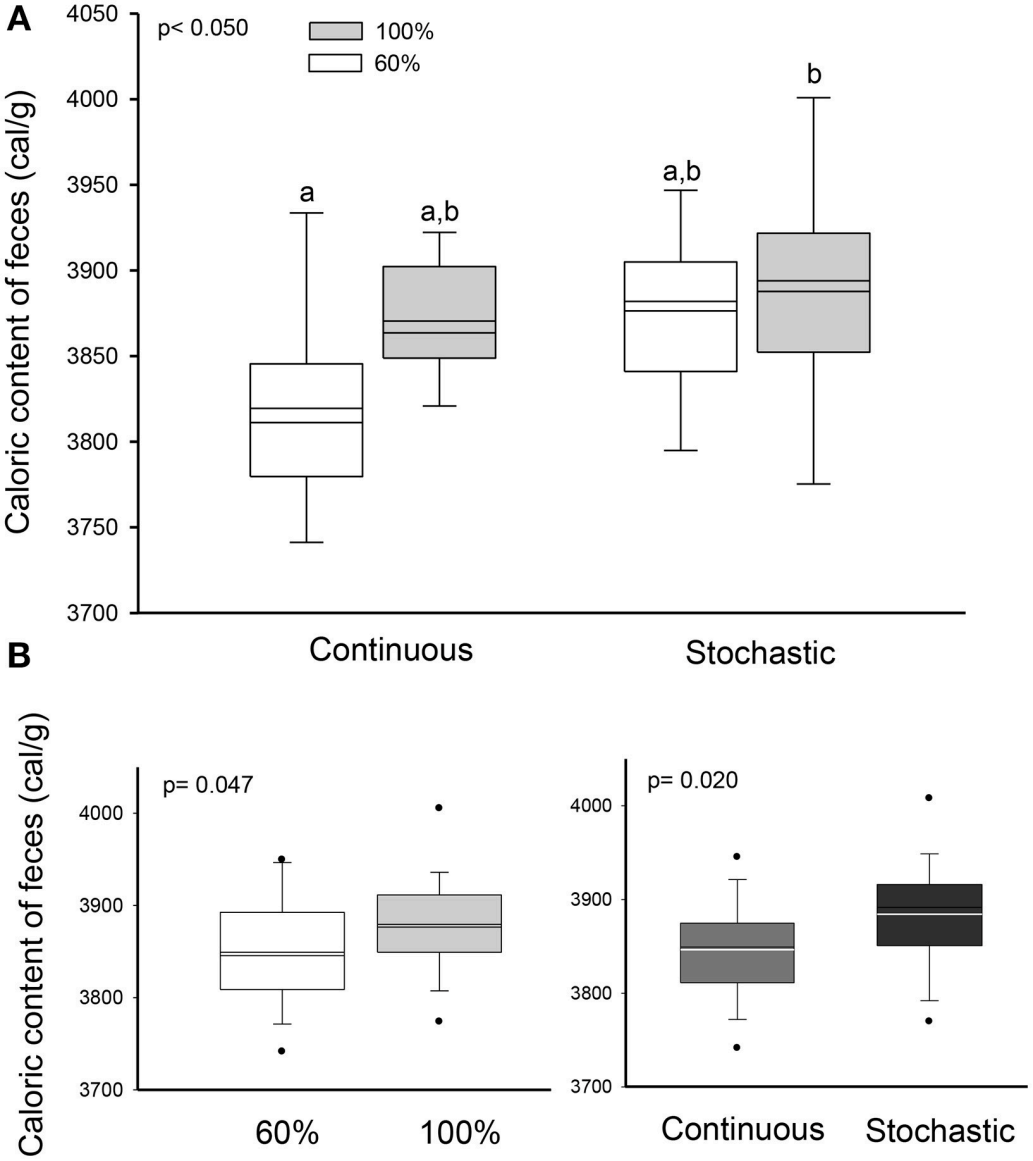
Effect of dietary regimens on the caloric content of feces (Factorial ANOVA), from adult males acclimated to four treatments; two types of caloric regimens (*ad libitum* and 60%); and two levels of periodicity of the regimens (continuous and stochastic treatment), for 20 days. **(A)** Comparison between treatments revealed that continuous treatment at 60% showed a significant lower caloric content per gram than the stochastic treatment *ad libitum* group. **(B)** Significant effect of the periodicity and caloric regimen type, in the caloric content but no effect on the interaction between these two factors.

We did not find a significant effect of the CR treatment on the activity of hydrolytic enzymes [ANOVA maltase: *F*_(3, 20)_ = 0.685, *p* = 0.572; sucrase: *F*_(3, 20)_ = 1.749, *p* = 0.189; and N-aminopeptidase *F*_(3, 20)_ = 0.378, *p* = 0.770, see Table 4). However, we observed a significant and positive correlation between the activity of intestinal n-aminopeptidases and the SCo of BrdU + cells (*r*^2^ = 0.514; *r* = 0.717; *p* = 0.009, Figure 6). Despite the fact that the analysis of energy content of feces revealed no differences at the beginning of the acclimation [*F*_(3, 43)_ = 0.889, *p* = 0.455], at the end of the experiments the C-60 group showed a significantly lower caloric content per gram than the S-100 group [*F*_(3, 43)_ = 3.726, *p* = 0.018, Figure 7]. In addition, we found a significant effect of the periodicity and caloric regimen type [factorial ANOVA *F*_(1, 43)_ = 5.800, *p* = 0.02; *F*_(1, 43)_ = 4.176, *p* = 0.047, respectively], but no effect on the interaction between these two factors [*F*_(1, 43)_ = 1.655, *p* = 0.205], where both stochastic and *ad libitum* treatments have the higher caloric content.

**Table 4:** Activity of cytochrome c oxidase from liver and intestinal digestive enzymes of *Mus musculus* acclimated to dietary regimens as indicated.

**Treatments**	**Cytochrome c oxidase**			**Maltase**	**Sucrase**	**n*-*aminopeptidase**
	**μmol/min**	**μmol/min g**	**μmol/min mg protein**	**UI mg^**−1**^ protein**	**UI mg^**−1**^ protein**	**UI mg^**−1**^ protein**
Continuous 60%	0.990 ± 0.709	0.664 ± 0.459	0.074 ± 0.061	0.0126 ± 0.003	0.003 ± 0.001	0.003 ± 0.001
Stochastic 60%	1.865 ± 1.083	1.276 ± 0.657	0.115 ± 0.065	0.015 ± 0.003	0.005 ± 0.002	0.003 ± 0.001
Continuous 100%	0.977 ± 0.678	0.652 ± 0.463	0.069 ± 0.076	0.015 ± 005	0.004 ± 0.002	0.003 ± 0.001
Stochastic 100%	0.952 ± 0.710	0.671 ± 0.399	0.053 ± 0.045	0.015 ± 0.005	0.003 ± 0.002	0.003 ± 0.001

### Proliferation Dynamics and Morphological Changes in the Intestinal Epithelium

**Figure 8:**
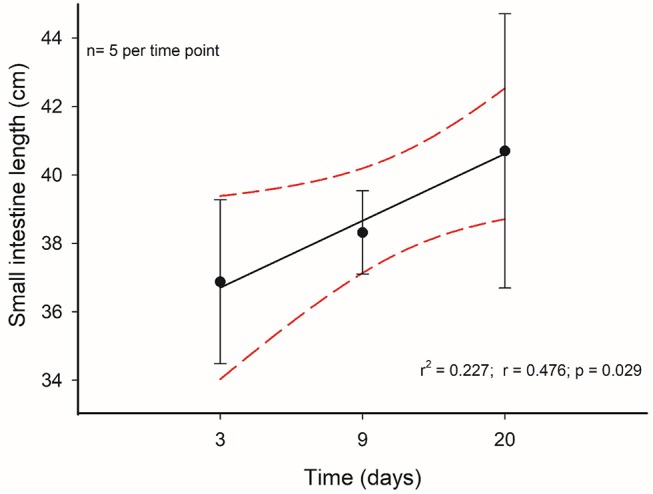
Time course of intestinal length change of mice who had a treatment with 60% of *ad libitum* intake. Segmented red lines indicate a 95% confidence interval.

**Figure 9:**
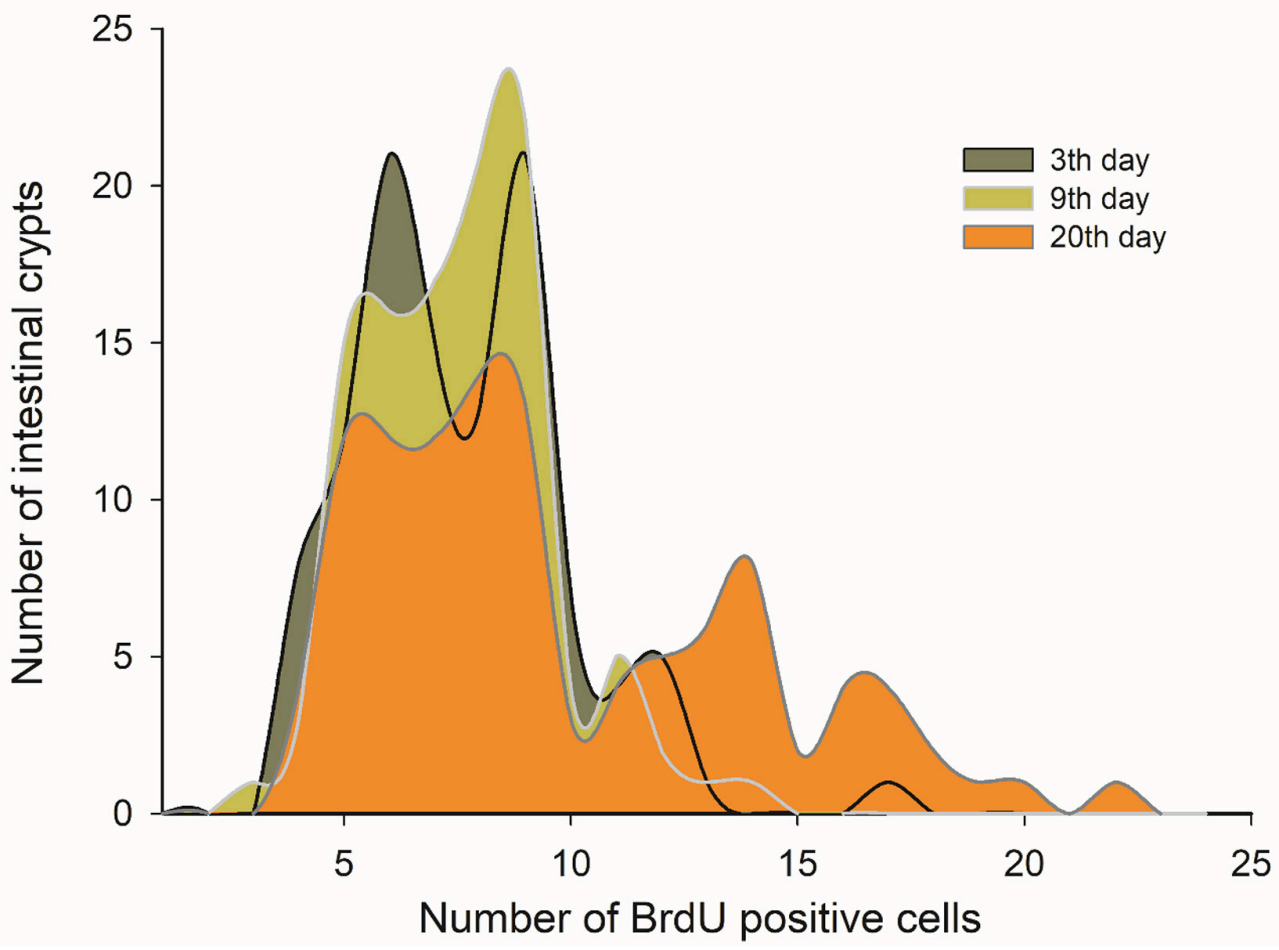
Histogram of absolute frequencies of intestinal crypts indicating distribution of BrdU+ cells. Adult males were acclimated at indicated times to a regimen of continuous feeding of caloric restriction at 60%.

Taking into account the above-mentioned results, we decided to analyze in more detail the morphological and histological features in individuals that received C-60 treatment by assessing at days 3, 9, and 20 of acclimation. Regarding the intestine length and mass, we did not find any significant differences between these groups [ANOVA *F*_(2, 17)_ = 0.859, *p* = 0.441]. Nevertheless, it is possible to recognize a gradual trend in the increment of the total length of the intestines along time. In fact, the intestine length associated positively with time: intestine length [cm] = 36.252 + 0.223^*^days (*r*^2^ = 0.227; *r* = 0.476; *p* = 0.029), with a growth rate of 0.223 cm per day in adult mice (Figure 8). This gradual change is predicted also by the SCo of BrdU+ cells, both for wet mass and residuals of length (*r*^2^ = 0.546; *r* = 0.739; *p* = 0.023 and *r*^2^ = 0.648; *r* = 0.805; *p* = 0.009, respectively). By analyzing the distribution of proliferating cells per crypts, we only found differences when comparing between the last day (day 20 of treatment) vs. days 3 and 9 (Kolmogorov-Smirnov *p* < 0.001; Figure 9).

### Metabolic Changes in the Liver

**Figure 10:**
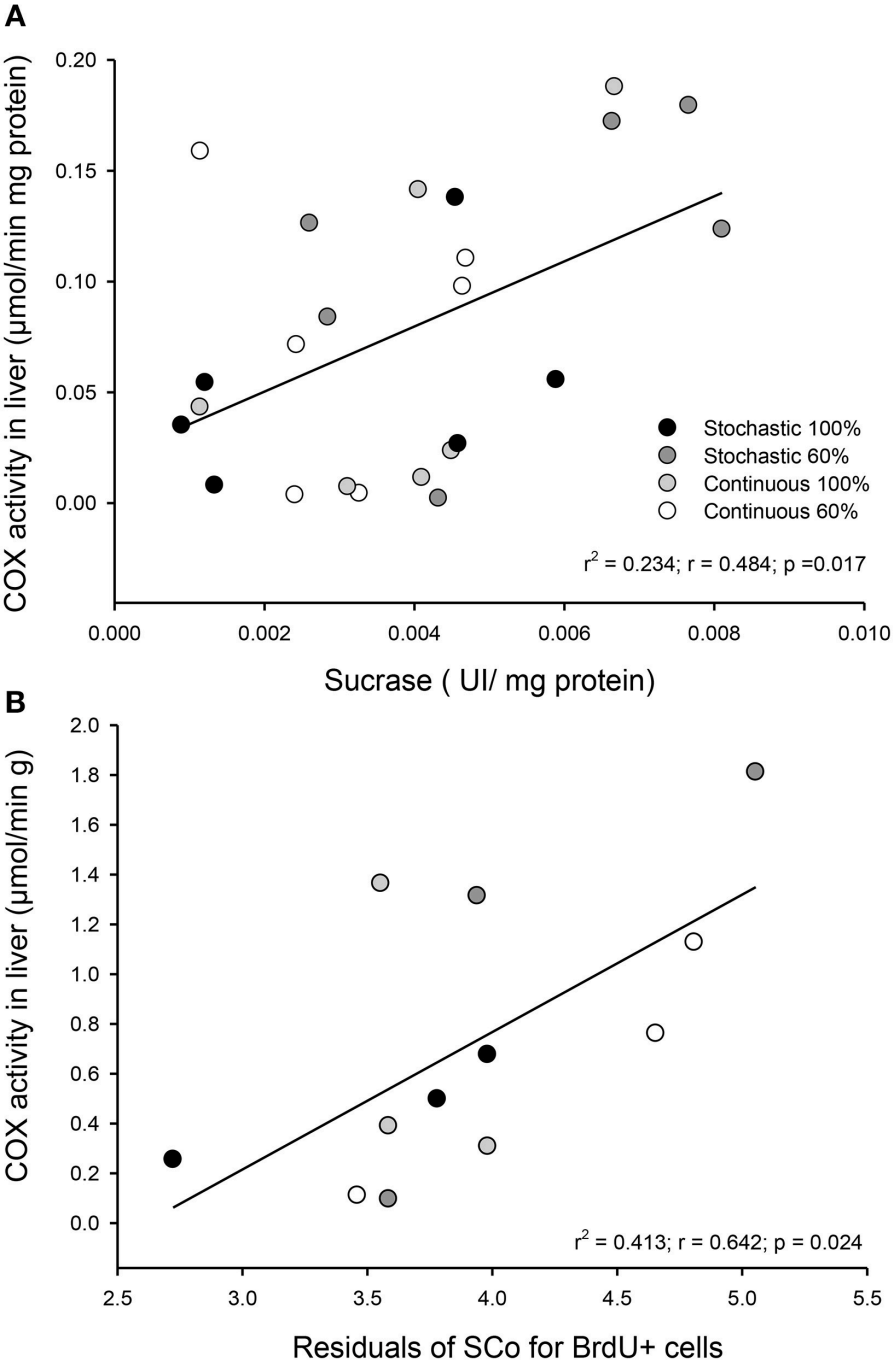
Observed relationship in *Mus musculus* individuals between liver metabolism and intestinal capacities after being under four different food treatments for 20 days. **(A)** Correlation between COX activity per milligram of protein in liver and the activity of sucrase in the first third of the intestine. **(B)** Correlation between COX activity per gram of tissue in liver and the coefficient of stabilization of the number of BrdU + cells per intestinal crypt.

Activity of COX in the liver was statistically indistinguishable among all groups [total activity: *F*_(3, 20)_ = 1.813, *p* = 0.177; per gram of tissue: *F*_(3, 20)_ = 2.221, *p* = 0.117; per milligram of protein: *F*_(3, 20)_ = 1.032, *p* = 0.400, Table 3]. Yet, we found a significant and positive relationship between the COX activity in the liver and the enzymatic activity of sucrase and the SCo for BrdU+ cells (*r*^2^ = 0.234; *p* = 0.017; *r*^2^ = 0.413; *p* = 0.024, respectively, Figure 10). Finally, we found a tendency toward a positive association between the activity of COX and the mass of the intestine (*r*^2^ = 0.153; *p* = 0.059).

### Rapamycin Treatment Could Mimic CR

CR has been shown to produce opposite regulation on mTORC1 in different intestinal cell types. While down-regulated in Paneth cells, mTORC1 is induced in intestinal SCs to drive an increase in cell number (Igarashi and Guarente, 2016), highlighting the importance of mTORC1 influencing intestinal homeostasis. Hence, we wondered if it might be possible to emulate the impact on proliferation that we observed with one of the dietary regimens by using the mTORC1 inhibitor Rapamycin for 20 days.

**Figure 11:**
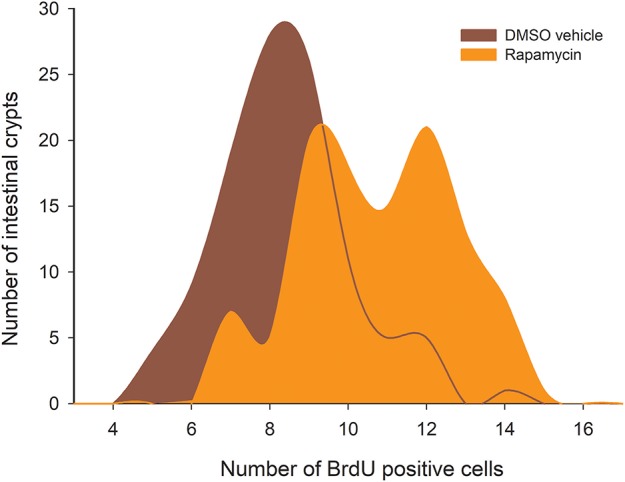
Histogram of absolute proliferative frequencies of intestinal crypts, comparing distribution of BrdU+ cells between the sham group (treated with DMSO), and Rapamycin treated animals. The analysis revealed statistical differences between both groups (Kolmogorov-Smirnov *p* < 0.001).

The analysis of the distribution of BrdU + cells revealed that both vehicle and drug treatments have only three peaks and are thus different to the ones displayed by the CR experimental groups (except for the comparison between S-60 and sham groups: D = 0.0926; *P* = 0.723). That said, we found significant differences between the Rapamycin group and the sham group (Kolmogorov-Smirnov *p* < 0.001, Figure 11). Notably, when analyzing the distribution of BrdU + cells in the Rapamycin group we found more crypts with a large number of proliferating cells, specifically, around 12–14 BrdU + cells/crypt. These results indicate that Rapamycin treatment most likely affected the SC's cell cycle, increasing the number of proliferating cells by crypt. The comparison of SCo indicate differences only between the Rapamycin treatment and both groups of 100%, i.e., C-100 and S-100 [ANOVA *F*_(5, 12)_ = 4.734, *p* = 0.013], revealing that inhibition of mTORC1 might mimic the CR *per-se*, rather than the stochasticity of the regimen.

## Discussion

The intestinal epithelium in mammals has a vigorously self-renewing capacity throughout the entire lifespan of an individual (Barker et al., 2010). As such, the structure of the small intestine has been advantageous for the analysis of developmental processes, SC biology, and integrative physiology. However, studies that evaluate processes of cell behavior and differentiation in a much broader context, for instance from an ecological or evolutionary perspective, are still scarce. To our knowledge, the present work is the first study that analyzes the effects of energy restriction and dietary stochasticity at both cellular and systemic levels in the same individual.

Within this context, our key finding here is that in environments with changing food availability and stochasticity, individuals exposed to a continuous and 60% food consumption regimen exhibit longer intestines along with a caloric reduction in the content of their feces. Moreover, mass and intestinal length, also morphological traits of the small intestine, were predicted strongly by the stabilization coefficient of BrdU+ cells per intestinal crypt, the latter correlating positively with the activity of n-aminopeptidases.

### Intestinal Morphological Changes Under CR

To date, most of the studies on SC proliferation and organ growth in the intestine have focused their attention in the context of ontogeny (Cripps and Williams, 1975; Fung et al., 2016; Meyer and Caton, 2016). In murids, small intestinal crypt development is initiated during the first postnatal week (Dehmer et al., 2011), and both crypt fission and crypt hyperplasia contribute to epithelial growth of the small intestine. The evidence seems to indicate that the peak of crypt fission occurs during milk feeding, whereas the peak of crypt hyperplasia occurs during weaning (Cummins et al., 2006). On the other hand, more recently, the impact of injury, inflammation or radiation on SC behavior within the base of the crypt has been characterized (Reddy et al., 2016; Schewe et al., 2016; Stzepourginski et al., 2017). Despite these data, so far knowledge about the intestinal dynamic under CR has been focused mainly on the understanding of signaling processes in SC within their niche (Yilmaz et al., 2012; Igarashi and Guarente, 2016), ignoring the broader physiological effects and their relationship with tissue changes and their environmental correlates.

In a first attempt to fill this gap, we aimed to understand whether and how SC plasticity contributes to differential CR responses in variable habitats. Our results indicate an increase of both the length and mass of the intestine, when rodents are acclimated for 20 days to a restricted diet consisting of 60% of the maximal consumption in a continuous feeding. Nevertheless, this result was not observed when feeding the animals in stochastic conditions. Zhao and Cao (2009) reported a similar result in *M. musculus* and *C. barabensis* subjected to food deprivation. However, Cao et al. (2009) and Zhu et al. (2014) found an increase in the length of intestines under stochastic food deprivation in *M. musculus* and *E. miletus*, respectively. Probably, the discrepancy among the results is due to differential responses between strains or species (e.g., we report only BALB/c strain responses), or the use of different acclimation protocols. Indeed, these studies used an *ad libitum* regimen in feeding days, in both cases, with different housing conditions (e.g., fresh saw dust bedding in animal cages or feeding with rabbit pellet chow).

In our experimental design, the animals have a high availability of food in days of no deprivation (see Table 1), allowing an incorporation of a greater amount of food. Thus, such condition may be physiologically perceived by the animals as a continued or *ad libitum* feeding, since there is always processed food in the gastrointestinal tract.

Regarding the processes explaining the increase of the mass and length of intestines, we found a relationship between these variables with the variation in SCo in the BrdU+ cells. That means that a longer intestine has less variability, in terms of the proliferative process occurring in its crypts. In other words, when an intestine increases its length, its crypts act more similarly (at least in the first third of the organ). Indeed, based on our results, only 20% of the length can be explained by the wet mass of the intestines. Thus, the stabilization coefficient of BrdU+ cells actually turns to be a better predictor, explaining 75% of the variability by mean SCo in the BrdU+ cells. In this vein, Parker et al. (2017) recently found a coupling between the proliferation in the crypts and the cell migration velocities along the villi. Taking into account this study, and the relation between Cleaved Caspase 3 and intestine length (Cleaved Caspase 3 + cells presented a positive relation with the intestine mass), we can hypothesize that rodents with longer intestines exhibit higher migration velocities along the villi. In addition, other studies reported that CR could affect SC behavior, producing an increase in the mean proliferative activity of SC in the crypts (Yilmaz et al., 2012). These findings contrast in part with our results, because our experimental groups did not display differences in the incorporation of BrdU, i.e., the percentage of BrdU + cells was the same among the different groups. Despite that, we developed a comparative analysis of the distribution of BrdU+ cells in the crypts (see Figure 4), revealing that the C-60 group, which had longer intestines, also exhibited a distinct distribution of these cells. As stated before, although we observed the presence of five peaks in the histograms of all experimental groups, there is an evident reduction in the first peak in the C-60 group with respect to all others, and a mayor frequency distribution of BrdU+ cells shifting to the 7–10, 12–14, and 15–20. We explain these peaks in terms of the tissue dynamics, considering that the normal growth and maintenance of intestinal tissue occurs through crypts fission by bifurcation and trifurcation of parental crypts (see Langlands et al., 2016). Thus, the first peak (accounting for proliferation of approximately seven cells) could represent normal or homeostatic proliferative processes, whereas the peaks around 14 and 20 BrdU + cells/crypt could represent crypts in fission by bifurcation and trifurcation, respectively. Considering the previous assumption, we propose that macroscopic variation in morphology, produced by CR, occurs through the reduction in the variability of proliferation between crypts (Synchronization), and an increment in the bifurcation and trifurcation processes. This increment both in the proliferation as well as in cell death (see similar result with Cleaved Caspase-3) coordination and increased number of crypts, could allow the extension of the intestine in a rate of approximately 0.2 cm per day.

Jackson (1915) studied the effect of acute and chronic fasting on organs mass of small mammals, demonstrating changes in the size of the digestive tract by inanition. But, as per our knowledge, our study is the first that evaluates the rate of change in the elongation of intestine in response to CR. Hence, to date, no data are available to compare with our results. However, based on the study of Zhao and Cao (2009) in Swiss mice (page 89, Table 2), we estimate the rate of change as ~0.2 cm per day, developed from a stochastic regimen to an *ad libitum* condition, with a reduction in the length. This fast growth capacity is well represented when epithelium wet masses increase rapidly by 40 to 75% in 1 day of refeeding in rats under long term starvation (phase III; Dunel-Erb et al., 2001).

Given the importance of knowing how SC respond to different environmental cues, and how their population dynamics are regulated, we analyzed the distribution of cells BrdU + in a treatment with the mTORC1 inhibitor Rapamycin. As previously reported by several studies (Blagosklonny, 2010; Yilmaz et al., 2012; Lee and Min, 2013), the inhibition of mTOR mimics CR. The novelty of our findings lies in that we found that Rapamycin actually modified the distribution of proliferating cells, and increased the coordination of those cells between crypts, a similar result to the one obtained by 60% caloric treatments (increase of the SCo). These findings seem to indicate the existence of a role of intestinal SC in the paradoxical phenomena of the intestine extension and, due to the dissimilarities in distribution profiles, suggest the participation of other molecular sensors (e.g., the other mTOR complex or SIRT proteins).

### Morphological Consequences of CR and Dietary Stochasticity in Other Organs

Interestingly, we found that the coordination of mitotic activity in the small intestine is related to an increase in the adipose tissue mass. An increment in the stomach mass has been reported in mice under CR when fed on daily basis (Hambly et al., 2007; Yang et al., 2007). Indeed, it has been proposed that chronic CR leads to a striking hypertrophy of lamina propria, stratum basale, stratum corneum, and the stratified squamous epithelium of the forestomach (Yang et al., 2007), the latter probably mediated by the ghrelin hormone (see Speakman and Mitchell, 2011).

The impact of experimental environmental food variability on non-digestive organs has received so far, less attention. Here, we found that the group of mice feeding *ad libitum* and continuously, exhibited a reduction in epididymal and inguinal adipose tissue, together with heavier mass reproductive organs and spleen. These results indicate that particularly this group reduces the generation of adipose reserves in an *ad libitum* feeding regime. However, our results contrast with similar studies in the field. For example, Bertrand et al. (1980) have shown that loss of white adipose tissue is disproportionately large under CR. In order to reconcile with previous studies, we propose that under our experimental design, animals acclimated to stochastics or CR conditions expressed an overcompensation in the accumulation of reserves during the feeding opportunities. Regarding the spleen mass, we found that the group of mice feeding *ad libitum* and continuously, exhibited lower masses than the group feeding continuously at 60% (i.e., CR). This difference is coincident with a reported reduction in the spleen size under CR (Yang et al., 2007). Thus, considering the negative relationship between the SCo of P-Histone 3 and the reproductive system and spleen mass, we suggest the existence of a trade-off between the coordination of intestinal mitotic activity and the reproductive and immune performance, a matter that deserves further research. Instead, we observe a positive relationship between BAT and the SCo of P-Histone 3, indicating that the generation of energy reserves are promoted by the coordination of intestinal mitotic activity.

### Digestive and Metabolic Effects

Recent data emphasize how the activity of metabolic pathways can enable intestinal SC to adapt to changing environments (Yilmaz et al., 2012; Igarashi and Guarente, 2016). Our results suggest that individuals of the C-60 group increased their digestive capacity, as feces had a lower energy content. This could be explained by improved changes in levels of reaction rates and absorption and/or by the elongation of the digestive organ. Regarding to reaction rates, we did not find differences in the disaccharidases activities among treatments. There is controversial information about the effect of CR or starvation on the levels of disaccharidases activities in mammals. For instance, previous findings have found a reduction in sucrase and maltase activities (61 and 80%, respectively) in rats after being exposed 5 days to starvation, while others reported an increase or even null effect upon jejunal lactase activity in starved rats (Holt and Yeh, 1992; Ihara et al., 2000). In line with our results, Raul et al. (1981) found that lactase activity was enhanced by starvation treatment, whereas sucrase activity showed no changes or decreased activity during the first 2 days of starvation. However, it is likely that the limited access to food can enhance the capacity to transport and absorb nutrients across the gastrointestinal tract without any increase in the levels of hydrolytic activities (Raul et al., 1981; Casirola et al., 1997; Ihara et al., 2000).

Even though disaccharidases activities remain constant among treatments, we found a strong and positive relation in the stabilization coefficient of BrdU+ cells per intestinal crypt + and the activity of n-aminopeptidases, suggesting that the reduction in the variability between crypts occurs in parallel to an increase in the capacity of protein digestion (see Figure 6). In these analyses, the highest activities and SCo index were found in individuals with a dietary condition of 60% restriction. Previous studies found an increment of n-aminopeptidases when measured after a few days of starvation in rats, reporting an increased activity after 2 days of treatment. Such results suggest that aminopeptidase may have a conserved role during the first days of starvation by preventing an important loss of tissue protein (Raul et al., 1981). Supporting this observation, Ihara et al. (2000) found that n-aminopeptidase and dipeptidyl peptidase IV activity were significantly elevated to 177 and 166%, respectively, during 5 days of starvation in rats.

The studies of the impact of CR on liver metabolism, conducted in both rats and mice, have identified changes at different levels, including in genomic profiling, increases in AMPK activity and increases of gluconeogenic and transaminase enzyme activities (Cao et al., 2001; Hagopian et al., 2003; Gonzalez et al., 2004). In spite of this, there are reports showing that the activities of glycolytic enzymes in liver (i.e., lactate dehydrogenase and pyruvate kinase) decrease under chronic CR in rats (Feuers et al., 1989). We found a positive association between the catabolic capacity of the liver and the sucrase activity in intestines. Besides, the hepatic metabolism, as revealed by COX activity, was higher in individuals exhibiting more synchronic proliferating crypts (Figure 10). These relationships indicate that both morphological and biochemical modifications in the small intestine, vary in tandem to the catabolic activity of the liver, suggesting that the intestinal modifications may promote the increase of ATP generating pathways in the liver (e.g., mitochondrial respiration). Interestingly, previous studies have reported that long term (i.e., 7–27 months) CR in both rats and mice increases the enzymatic activities related to the gluconeogenesis capacity of the liver along with a decrease of the enzymatic capacity for glycolysis (Dhahbi et al., 1999; Cao et al., 2001). Thus, the catabolic capacity of liver appears to adjust to changes in nutrient intake, as a function of the extension of the dietary alteration. Given this plasticity, the liver metabolism could be a key factor in the attempt to understand the physiological responses to the CR and environmental dietary variability, and ultimately help to understand processes such as increased lifespan, energy management and savings (e.g., torpor), and the generation of the so-called “paradoxical responses” when animals are faced with CR (Holmes and Mistlberger, 2000; Cao et al., 2009) namely the allocation of energy and biomolecules to the increment of an organ, despite the reduced food.

In summary, as has been previously demonstrated in both laboratory and wild small mammals, species are able to modify their digestive traits at different levels of biological integration to changes in both environmental (e.g., diet and temperature) and internal conditions (e.g., reproductive state, parasites). Our results indicate for the first time that CR induces changes in the level of cell proliferation dynamics (i.e., crypt coordination and possibly increased fission) *in vivo*, that is, in their native SC niche. Hence, our data for now allow us to explain the morphological and functional changes in the small intestine of this particular murine model. We suggest that the phenomenon of rapid intestine plasticity could be explained by mechanisms in which cell proliferation and tissue turnover are balanced in order to yield positive consequences on food processing capacities and metabolic performance. Therefore, the phenomena analyzed could constitute a short-term mechanism that increases an animal's fitness in a variable environmental context.

### Future Perspectives

It has been widely documented that animals adjust their digestive attributes (e.g., mass and/or length) to changes in food availability and dietary quality to maximize overall energy return (Naya et al., 2008). Thus, the development of a similar analysis as the one presented here, could reveal mechanisms of tissue dynamics, such as coordination of proliferation in intestinal crypts. Indeed, these phenomena could be explored in other cases of intestinal plasticity previously reported, both in ecological studies (for reviews, see Karasov and Diamond, 1983; McWilliams and Karasov, 2001; Naya and Bozinovic, 2004; Naya, 2008) and biomedical research (Mihaylova et al., 2014; Dayton and Clevers, 2017).

Additionally, in future studies the intestinal changes observed in this work could be related to other physiological phenomena associated with CR. Metabolic and thermoregulatory changes, behavioral exploration, and extension of life span in rodents (Colman et al., 2014; Sohal and Forster, 2014; Lusseau et al., 2015; Mitchell et al., 2016; Yamada et al., 2017), as well as studies exploring the impact of certain nutrients in the differentiation of intestinal SC, as recently reported in *Drosophila melanogaster* (Obniski et al., 2018), may contribute to elucidate how diet-induced cellular changes impact aging and diseases.

## Author Contributions

IP-V, PS, and VP designed research. IP-V, IC-M, and PL performed research. IP-V and IC-M analyzed data. IP-V, PS, and VP wrote the paper.

### Conflict of Interest Statement

The authors declare that the research was conducted in the absence of any commercial or financial relationships that could be construed as a potential conflict of interest.
